# Home-Based and Facility-Based Directly Observed Therapy of Tuberculosis Treatment under Programmatic Conditions in Urban Tanzania

**DOI:** 10.1371/journal.pone.0161171

**Published:** 2016-08-11

**Authors:** Francis Mhimbira, Jerry Hella, Thomas Maroa, Shadrack Kisandu, Magreth Chiryamkubi, Khadija Said, Grace Mhalu, Abdallah Mkopi, Beatrice Mutayoba, Klaus Reither, Sébastien Gagneux, Lukas Fenner

**Affiliations:** 1 Ifakara Health Institute, Dar es Salaam, Tanzania; 2 Swiss Tropical and Public Health Institute, Basel, Switzerland; 3 University of Basel, Basel, Switzerland; 4 National TB and Leprosy Programme, Ministry of Health, Community Development, Gender, Elderly and Children, Dar es Salaam, Tanzania; 5 Institute of Social and Preventive Medicine, University of Bern, Bern, Switzerland; Indian Institute of Science, INDIA

## Abstract

**Introduction:**

Decentralization of Directly Observed Treatment (DOT) for tuberculosis (TB) to the community (home-based DOT) has improved the coverage of TB treatment and reduced the burden to the health care facilities (facility-based DOT). We aimed to compare TB treatment outcomes in home-based and facility-based DOT under programmatic conditions in an urban setting with a high TB burden.

**Methodology:**

A retrospective analysis of a cohort of adult TB patients (≥15 years) routinely notified between 2010 and 2013 in two representative TB sub-districts in the Temeke district, Dar es Salaam, Tanzania. We assessed differences in treatment outcomes by calculating Risk Ratios (RRs). We used logistic regression to assess the association between DOT and treatment outcomes.

**Results:**

Data of 4,835 adult TB patients were analyzed, with a median age of 35 years, 2,943 (60.9%) were men and TB/HIV co-infection prevalence of 39.9%. A total of 3,593 (74.3%) patients were treated under home-based DOT. Patients on home-based DOT were more likely to die compared to patients on facility-based DOT (RR 2.04, 95% Confidence Interval [95% CI]: 1.52–2.73), and more likely to complete TB treatment (RR 1.14, 95% CI: 1.06–1.23), but less likely to have a successful treatment outcome (RR 0.94, 95% CI: 0.92–0.97). Home-based DOT was preferred by women (adjusted Odds Ratio [aOR] 1.55, 95% CI: 1.34–1.80, p<0.001), older people (aOR 1.01 for each year increase, 95% CI: 1.00–1.02, p = 0.001) and patients with extra-pulmonary TB (aOR 1.45, 95% CI: 1.16–1.81, p = 0.001), but less frequently by patients on a retreatment regimen (aOR 0.12, 95% CI: 0.08–0.19, p<0.001).

**Conclusions/significance:**

TB patients under home-based DOT had more frequently risk factors of death such as older age, HIV infection and sputum smear-negative TB, and had higher mortality compared to patients under facility-based DOT. Further operational research is needed to monitor the implementation of DOT under programmatic conditions.

## Introduction

In 2014 globally, almost 1.5 million people died from tuberculosis (TB) from an estimated 9.6 million who developed TB [1]. TB is now the leading cause of death from an infectious disease worldwide, surpassing those caused by Human Immunodefiency Virus [2]. Globally, TB mortality trends are on the decline [1], but remain high despite effective short-course treatment regimens [1]. In Africa, however, the decline did not meet the 2015 Stop TB Partnership goal of a 50% decline from 1990 to 2015 [1].

Early diagnosis and effective treatment of TB are critical to reduce TB mortality and control the spread of TB [1]. The Directly Observed Treatment Short course (DOTS) strategy recommended by the World Health Organization in 1994 [3–5], has proved to be one of the most effective public health interventions [5]. The DOTS strategy provides a comprehensive package to control TB which consists of six components, and one of them addresses the use of standardized treatment with supervision and patients supporters (directly observed therapy, DOT) [4]. DOT was pioneered in Tanzania in the 1980s, and resulted in improved cure rates from 60% to 80% [5]. TB treatment under DOT can be given at the health facility (facility-based DOT) or in the community (home-based DOT). The facility-based DOT approach requires that patients visit daily the health facility for supervised drug intake by health workers, with continuous assessment of adherence to TB medication [6]. However, this delivery system places a burden on the health care system and the patient [7,8]. This made it necessary to decentralize TB treatment to the community (home-based DOT) [8,9].

Although systematic reviews showed that patients under home-based DOT can achieve similar or better treatment outcomes compared to facility-based DOT [10,11], the implementation of treatment under home-based DOT has also raised concerns [12]. Health care workers have expressed concerns about treatment adherence, storage of drugs and lack of supervision under home-based DOT which may contribute to unfavorable TB treatment outcomes [13]. We therefore aimed to assess TB treatment outcomes in home-based and facility-based DOT under programmatic conditions in the high TB incidence country Tanzania.

## Methods

### Ethics statement

We used anonymized population-level data of notified TB patients. Hence, ethical approval and informed consent were not required for this analysis. The permission to use the data was authorized by the Program Manager of the National TB and Leprosy Programme (NTLP) in the Ministry of Health, Community Development, Gender, Elderly and Children (MoHCDGEC). All TB patients received the standardized TB treatment regimen in line with national TB treatment guideline [6]. HIV-positive TB patients were managed in accordance to the National Guidelines for the Management of HIV in Tanzania [14].

### Study setting

Temeke is one of the three districts in Dar es Salaam region in Tanzania. Temeke is the largest district that occupies 48.3% of the total surface area of Dar es Salaam and has a total population of 1,368,881 [15,16]. Temeke district notified 4,373 TB patients in 2014 [17] and has HIV prevalence of 5.2% in the general adult population [16]. We selected Temeke district because it represents an urban setting with a high TB notification rate, and because it was one of the few districts in Tanzania where the acceptability study of home-based and facility-based DOT was done in 2006 [7]. The two sub-districts of Wailes I and Wailes II, as categorized by National TB and Leprosy Programme, are densely populated areas surrounding the Temeke district hospital which has the largest TB clinic serving these two sub-districts.

### Study population and study definitions

**Fig 1:**
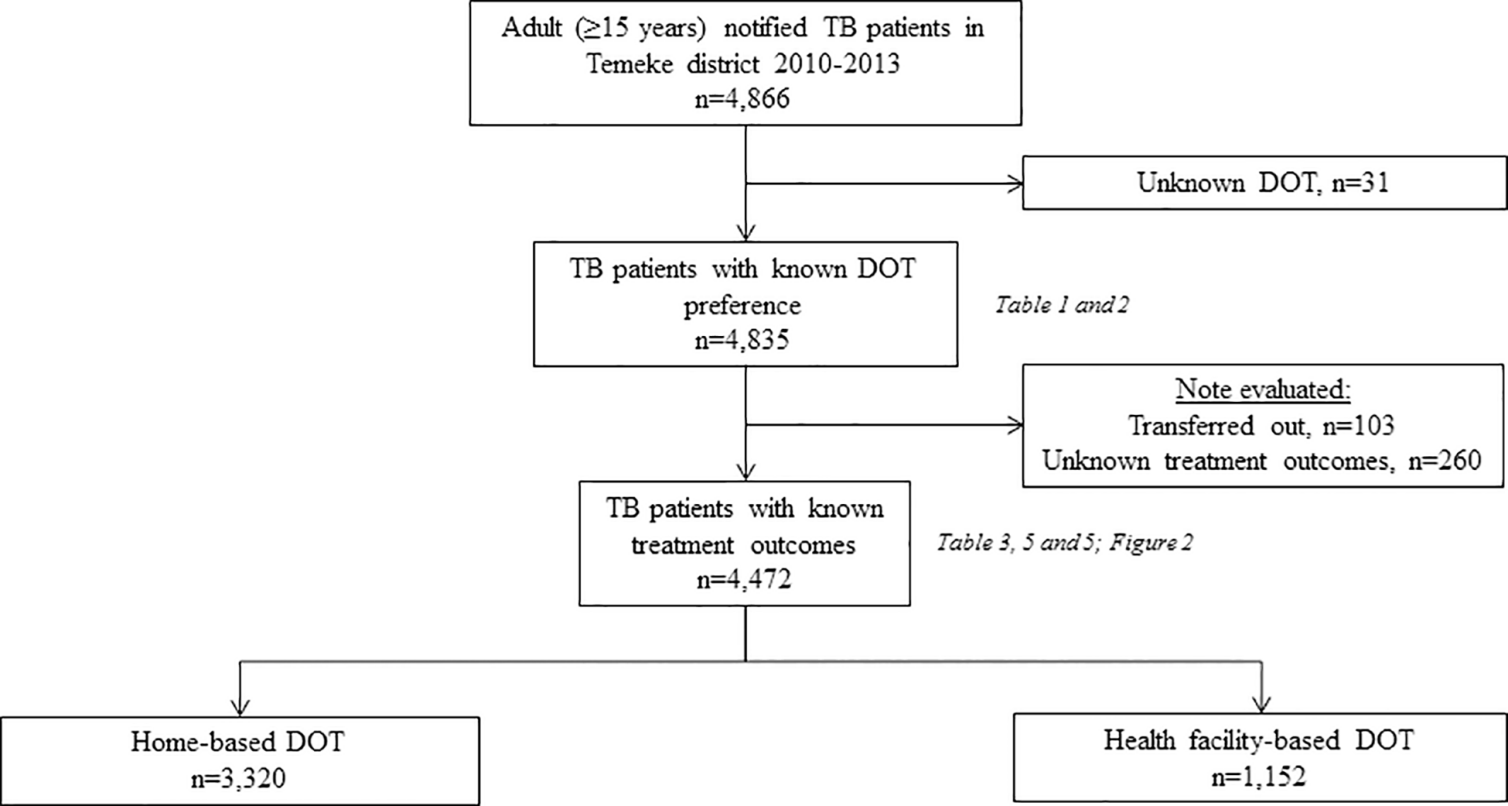
Selection of patients included in the study.

We obtained anonymized electronic data from the TB district register of all adult TB patients (aged ≥15 years) who were notified between 2010 and 2013 in a single geographical area of two TB sub-districts (Wailes I and Wailes II) in the Temeke district, Dar es Salaam, Tanzania. The patient selection is presented in Fig 1. We excluded 31 patients with unknown DOT preference and 363 patients with treatment outcome “not evaluated” (“unknown” or “transferred out”). The included and excluded (patients with unknown DOT and “not evaluated” outcome) patients did not differ in baseline characteristics (S1 Table).

We used the study definitions according to the WHO updated guidelines [18]. A “new” patient was defined as never had treatment for TB, or has taken anti-TB drugs for less than one month; a “retreatment” patient was defined as previously treated patient who has received one month or more of anti-TB drugs in the past; TB death as any death that occurred during TB treatment. “Treatment success” combines cured and treatment completed outcomes [18]. “Unsuccessful” treatment combines failed treatment, loss to follow-up and died outcomes. Not evaluated combines the transferred-out and unknown treatment outcomes.

### Facility-based and home-based DOT

According to the national guidelines, facility-based DOT requires health care workers to supervise daily intake of anti-TB medication during the intensive and continuation phases [6]. While at the clinic, TB patients will also receive health education and clinical evaluation by the clinicians and nurses. Under home-based DOT, a patient takes medication at home and is supervised by a treatment supporter who is chosen by the TB patient [6]. The treatment supporter, who can be either a family member or a neighbor, supervises anti-TB medication intake at home on daily basis during the entire TB treatment phase [6,13]. Treatment supporters would also accompany the TB patients to the health facility for anti-TB medication refill weekly during the intensive phase and fortnightly during the intensive phase. TB patients with their treatment supporters while at the health facility will receive health education from health care workers; and TB patients will undergo a clinical evaluation [6]. Under the current Patient-Centered Treatment (PCT) approach, the patient can indicate the choice of DOT at the time of TB diagnosis (hereafter referred as “preference of DOT”).

### Laboratory investigations

TB diagnosis was made either by microbiological confirmation of acid fast bacilli (AFB) in the sputum smear examination by Ziehl-Nielsen method or based on clinical grounds or radiological findings as determined by the attending clinician at the TB clinic. The quality control of smear microscopy was done by the Central Tuberculosis Reference Laboratory (CTRL) in Dar es Salaam. AFB smear positive results was as per World Health Organization/International Union Against Tuberculosis and Lung Disease grading: “scanty” with of 1–9 AFB per 100 oil immersion fields; 1+” with 10–99 AFB per 100 immersion fields; “2+” with 1–10 AFB per 1 immersion field and “3+” with >10 AFB per immersion field [6,19].Tanzania also implements collaborative TB/HIV services, and all TB patients were offered rapid HIV testing as per National guidelines for the management of HIV and AIDS of Tanzania [14].

### Statistical analyses

Descriptive statistics were used for patient characteristics, and groups were compared using Chi-Square and Wilcox rank-sum test as appropriate. We estimated treatment outcomes risk ratios (RR) comparing patients under home-based with patients under health facility-based DOT. We used logistic regression models to estimate the effect of home-based DOT on mortality and treatment success. The results are presented as crude and adjusted odds ratio (OR) after adjusting for age, sex, HIV status, site of disease, AFB sputum smear results at diagnosis, and patient category (new or retreatment patient). Point estimates are presented with their corresponding 95% Confidence Intervals (95% CIs). We also performed a complete case analysis excluding all patients with any missing value. All analyses were performed using Stata version 14.0 (Stata Corp, College Station, TX, USA).

## Results

### Patient characteristics

We analyzed 4,835 adult TB patients from the Temeke District, Dar es Salaam, notified between 2010 and 2013. The median age was 35 years (Interquartile Range [IQR]: 27–44 years); 2,943 (60.9%) were men. The HIV prevalence was 39.9% (95% Confidence Interval [95% CI]: 38.5–41.3%). Home-based DOT was the most preferred (3,593 patients, 74.3%) compared to facility-based DOT. Patients on home-based DOT were more likely to be HIV-positive, women, with EPTB, smear-negative at diagnosis and older (Table 1). TB/HIV co-infected patients were more frequently men, of older age, and patients with EPTB and smear-negative TB, and opted more often for home-based DOT (S2 Table).

**Table 1:** Baseline characteristics of TB patients at Temeke district by choice of DOT.

Characteristics	All	Home-based	Facility-based	p-value
	(n = 4,835)	(n = 3,593)	(n = 1,242)	
	n (%)	n (%)	n (%)	
**Proportion of diagnosis, n (%)**	4,835 (100)	3,593 (100)	1,242 (100)	
**Sex**				<0.001
Male	2,943 (60.9)	2,082 (57.9)	861 (69.3)	
Female	1,892 (39.1)	1,511 (42.1)	381 (30.7)	
**Age in years, median (IQR)**	35 (27–44)	35 (29–45)	34 (28–41)	0.007^a^
**Age groups (years)**				<0.001
15–19	285 (5.9)	238 (6.6)	47 (3.8)	
20–24	544 (11.3)	390 (10.9)	154 (12.4)	
25–29	724 (15.0)	521 (14.5)	203 (16.3)	
30–34	820 (17.0)	577 (16.1)	243 (19.6)	
35–39	706 (14.6)	521 (14.5)	185 (14.9)	
40–44	570 (11.8)	405 (11.3)	165 (13.3)	
45–49	428 (8.9)	319 (8.9)	109 (8.8)	
50–54	279 (5.8)	218 (6.1)	61 (4.9)	
≥55	479 (9.9)	404 (11.2)	75 (6.0)	
**HIV status**				0.002
Positive	1,927 (39.9)	1,485 (41.3)	442 (35.6)	
Negative	2,582 (53.4)	1,874 (52.2)	708 (57.0)	
Unknown status	326 (6.7)	234 (6.5)	92 (7.4)	
**Site of disease**				<0.001
PTB	3,977 (82.3)	2,884 (80.3)	1,093 (88.0)	
EPTB	858 (17.7)	709 (19.7)	149 (12.0)	
**Category**				<0.001
New	4,706 (97.3)	3,557 (99.0)	1,149 (92.5)	
Retreatment	129 (2.7)	36 (1.0)	93 (7.5)	
**AFB smear result at diagnosis**				<0.001
Smear-positive	2,438 (50.4)	1,706 (47.5)	732 (58.9)	
Smear-negative	2,352 (48.6)	1,845 (51.3)	507 (40.8)	
Unknown	47 (0.9)	42 (1.2)	3 (0.2)	

^a^Wilcox-ranksum test; n (%), absolute number and column percentage

Patients with unknown DOT preference were excluded (n = 31, 0.6%).

AFB, acid-fast bacilli; IQR, Interquartile Range; HIV, Human Immunodeficieny Virus; TB, Tuberculosis; PTB, Pulmonary tuberculosis; EPTB, Extrapulmonary tuberculosis; DOT, Directly Observed Treatment.

### TB treatment outcomes by DOT preference

Overall, treatment success which combines cured and treatment completed outcomes, was reported in 4,006 (82.8%) patients and 345 (7.1%) patients died during TB treatment. Patients on home-based compared to facility-based DOT were less likely to have treatment success (RR 0.94, 95% CI: 0.92–0.97). We observed no significant difference in loss to follow-up and “not evaluated” treatment outcomes. Treatment failure was only reported in the home-based DOT (24 patients, 0.7%). When restricting the analysis to smear-positive TB patients, patients on home-based DOT were less likely to be cured compared to facility-based DOT (RR, 0.93 95 CI: 0.88–0.98), and were more likely to die but the results were not significant (RR 1.35, 95% CI: 0.88–2.06). We found no statistically significant differences in all other treatment outcomes (Table 2). Of 31 TB patients with unknown DOT preference, 2 patients died during the TB treatment.

**Table 2:** Differences in TB treatment outcomes among TB patients under home-based DOT compared to facility-based DOT.

Treatment outcome	All	Home-based DOT	Facility-based DOT	RR (95% CI)	p-value
	n (%)	n (%)	n (%)		
**All patients**	4,835	3,593 (100)	1,242 (100)		<0.001
Treatment success	4,006 (82.8)	2,932 (81.6)	1,073 (86.4)	0.94 (0.92–0.97)	
Cured	1,738 (35.9)	1,192 (33.2)	546 (44.0)	0.75 (0.70–0.82)	
Treatment completed	2,268 (46.9)	1,741 (48.5)	527 (42.4)	1.14 (1.06–1.23)	
Died	345 (7.1)	295 (8.2)	50 (4.0)	2.04 (1.52–2.73)	
Loss to follow-up	97 (2.0)	68 (1.9)	29 (2.3)	0.81 (0.53–1.25)	
Treatment failed	24 (0.5)	24 (0.7)	0 (0)	ND	
Not evaluated	363 (7.5)	273 (7.6)	90 (7.2)	1.05 (0.83–1.32)	
Smear-positive	2,438 (100)	1,706 (100)	732 (100)		0.024
**Treatment success**	2060 (84.5)	1431 (83.9)	629 (85.9)	0.98 (0.94–1.01)	
Cured	1,738 (71.3)	1,192 (69.9)	546 (74.6)	0.93 (0.88–0.99)	
Treatment completed	322 (13.2)	239 (14.0)	83 (11.3)	1.23 (0.98–1.56)	
Died	109 (4.5)	83 (4.9)	26 (3.6)	1.35 (0.88–2.06)	
Loss to follow-up	52 (2.1)	34 (2.0)	18 (2.5)	0.83 (0.48–1.46)	
Treatment failed	14 (0.6)	14 (0.8)	0 (0)	ND	
Not evaluated	203 (8.3)	144 (8.4)	59 (8.1)	1.04(0.78–1.40)	

Patients with unknown DOT preference were excluded (n = 31, 0.6%; n (%) = absolute number and column percentage

DOT, Directly Observed Treatment; RR, Risk ratio; 95% CI: 95% Confidence Interval; ND, Not Defined; Not evaluated; Transferred out and unknown TB treatment outcome.

### TB mortality and associated patient factors

TB mortality was strongly associated with home-based compared to facility-based DOT (adjusted OR [aOR] 2.28, 95% CI: 1.64–3.18, p<0.001). Other patient factors associated with mortality included older age (e.g ≥45 years, aOR 2.05, 95% CI: 1.36–3.08, p<0.002), HIV-positive (aOR 1.68, 95% CI: 1.31–2.15, p<0.001), retreatment category (aOR 3.99, 95% CI: 2.33–6.84, p<0.001) and smear-positive TB (aOR 0.49, 95% CI: 0.38–0.64, p<0.001) (Table 3). A complete case analysis (excluding any missing values) produced similar results. S3 Table shows the patient characteristics separately for home-based and facility-based DOT for TB patients who were reported as dead or alive.

**Table 3:** Risk factors for mortality among TB patients.

Characteristic	Crude OR		Adjusted OR	
	OR (95% CI)	p-value	OR (95% CI)	p-value
**Place of DOT**		<0.001		<0.001
Facility	1 (Ref)		1(Ref)	
Home	2.15 (1.58–2.92)		2.28 (1.64–3.18)	
**Sex**		0.2		0.8
Male	1 (Ref)		1 (Ref)	
Female	1.14 (0.92–1.43)		0.97 (0.77–1.23)	
**Age groups (years)**		<0.001		0.002
<25	1 (Ref)		1 (Ref)	
25–34	1.47 (0.97–2.22)		1.37 (0.90–2.08)	
35–44	2.15 (1.44–3.22)		1.69 (1.11–2.57)	
≥45	2.64 (1.78–3.93)		2.05 (1.36–3.08)	
**HIV status**		<0.001		<0.001
Negative	1 (Ref)		1 (Ref)	
Positive	2.07 (1.65–2.61)		1.68 (1.31–2.15)	
Unknown HIV status	1.11 (0.68–1.82)		0.98 (0.59–1.63)	
**Site of disease**		0.006		0.8
PTB	1 (Ref)		1 (Ref)	
EPTB	1.46 (1.12–1.89)		0.93 (0.59–1.63)	
**AFB smear result at diagnosis**		<0.001		<0.001
Smear-negative	1 (Ref)		1 (Ref)	
Smear-positive	0.44 (0.35–0.56)		0.49 (0.38–0.64)	
Unknown	1.82 (0.80–4.17)		1.39 (0.60–3.25)	
**Category of TB patient**		0.001		<0.001
New	1 (Ref)		1 (Ref)	
Retreatment	2.61 (1.59–4.29)		3.99 (2.33–6.84)	

OR, Odds Ratio; 95% CI = 95% Confidence Interval; DOT, Directly Observed Treatment; Ref, Reference Group; HIV, Human Immunodeficiency Virus; TB, Tuberculosis; PTB, Pulmonary tuberculosis; EPTB, Extra-pulmonary tuberculosis

All variables were included in the adjusted model

Excluded from the analysis: Not evaluated = Transferred-out and TB patients with unknown TB treatment outcomes, n = 363, 7.5%.

### Successful treatment outcome and associated patient factors

Treatment success was less likely to be associated with home-based compared to facility-based DOT (aOR 0.53, 95% CI: 0.41–0.70, p<0.001). Treatment success was less likely with increasing age, among HIV-positive and patients on retreatment category and smear-negative TB patients (Table 4).

**Table 4:** Factors associated with treatment success among adult TB patients.

Characteristic	Crude OR		Adjusted OR	
	OR (95% CI)	p-value	OR (95% CI)	p-value
**Place of DOT**		<0.001		<0.001
Facility-based	1 (Ref)		1 (Ref)	
Home-based	0.56 (0.43–0.72)		0.53 (0.41–0.70)	
**Sex**		0.9		0.2
Male	1 (Ref)		1 (Ref)	
Female	1.0 (0.82–1.22)		1.15 (0.94–1.41)	
**Age groups (years)**		<0.001		0.03
<25	1 (Ref)		1 (Ref)	
25–34	0.79 (0.57–1.09)		0.84 (0.60–1.17)	
35–44	0.60 (0.43–0.83)		0.73 (0.52–1.03)	
≥45 years	0.51 (0.37–0.70)		0.63 (0.45–0.88)	
**HIV status**		<0.001		<0.001
Negative	1 (Ref)		1 (Ref)	
Positive	0.55 (0.45–0.67)		0.62 (0.50–0.77)	
Unknown HIV status	0.76 (0.51–1.12)		0.81 (0.54–1.20)	
**Site of disease**		0.046		0.7
PTB	1 (Ref)		1 (Ref)	
EPTB	0.79 (0.62–0.99)		1.09 (0.84–1.41)	
**AFB smear results at diagnosis**		<0.001		<0.001
Smear-negative	1 (Ref)		1 (Ref)	
Smear-positive	1.74 (1.43–2.12)		1.61 (1.29–2.01)	
Unknown	0.59 (0.27–1.29)		0.74 (0.33–1.64)	
**Category of TB patient**		0.02		<0.001
New	1 (Ref)		1 (Ref)	
Retreatment	0.45 (0.28–0.72)		0.34 (0.21–0.56)	

OR, Odds Ratio; 95% CI = 95% Confidence Interval; DOT, Directly Observed Treatment; Ref, Reference Group; HIV, Human Immunodeficiency Virus; TB, Tuberculosis; PTB, Pulmonary tuberculosis; EPTB, Extra-pulmonary tuberculosis

All variables were included in the adjusted model

Excluded: Not evaluated = Transferred-out and TB patients with unknown TB treatment outcomes, n = 363, 7.5%.

### Patient factors associated with preference of home-based DOT

Patients opting for home-based DOT were more likely to be women (aOR 1.55, 95% CI: 1.34–1.80, p<0.001), older (aOR 1.01, 95% CI: 1.00–1.02, p = 0.001; for each year increase in age), more likely to have EPTB (aOR 1.45, 95% CI: 1.16–1.81, p<0.001), but less likely to have smear-positive TB (aOR 0.80, 95% CI: 0.68–0.93, p = 0.002) and less likely to be on a retreatment category (aOR 0.12, 95% CI: 0.08–0.19, p<0.001). HIV-positive TB patients tended to prefer home-based compared to facility-based DOT (aOR 1.16, 95% CI: 0.99–1.36, p = 0.052) (Table 5).

**Table 5:** Factors associated with preference to home-based DOT.

Characteristic	Crude OR		Adjusted OR	
	OR (95% CI)	p-value	OR (95% CI)	p-value
**Sex**		<0.001		<0.001
Male	1 (Ref)		1 (Ref)	
Female	1.63 (1.41–1.88)		1.55 (1.34–1.80)	
**Age groups (years)**		<0.001		<0.001
<25	1 (Ref)		1 (Ref)	
25–34	0.78 (0.63–0.95)		0.73 (0.59–0.90)	
35–44	0.83 (0.67–1.02)		0.77 (0.61–0.96)	
≥45 years	1.24 (0.99–1.54)		1.17 (0.93–1.48)	
**HIV status**		0.005		0.05
Negative	1 (Ref)		1 (Ref)	
Positive	1.24 (1.08–1.43)		1.16 (0.99–1.36)	
Unknown HIV status	0.92 (0.71–1.19)		0.86 (0.66–1.13)	
**Site of disease**		<0.001		0.001
PTB	1 (Ref)		1 (Ref)	
EPTB	1.81 (1.49–2.20)		1.43 (1.14–1.78)	
**AFB smear results at diagnosis**		<0.001		0.001
Smear negative	1 (Ref)		1 (Ref)	
Smear positive	0.64 (0.56–0.74)		0.78 (0.69–0.92)	
Unknown smear result	3.41 (1.04–11.11)		3.38 (1.02–11.23)	
**Category of TB patient**		<0.001		<0.001
New TB patient	1 (Ref)		1 (Ref)	
Retreatment	0.13 (0.08–0.19)		0.12 (0.08–0.19)	

OR, Odds Ratio; 95% CI = 95% Confidence Interval; DOT, Directly Observed Treatment; Ref, Reference Group; HIV, Human Immunodeficiency Virus; TB, Tuberculosis; PTB, Pulmonary tuberculosis; EPTB, Extra-pulmonary tuberculosis

EPTB, Extra-pulmonary Tuberculosis

All variables were included in the adjusted model

n = 4,472; Excluded: Not evaluated = Transferred-out and TB patients with unknown TB treatment outcomes, n = 363, 7.5%.

## Discussion

We showed that TB patients who opted for home-based DOT had more frequently risk factors for mortality and a higher mortality compared to the facility-based DOT group. The risk factors for mortality were older age (>35 years), HIV infection, smear-negative TB, and being on a TB retreatment regimen.

Our main finding demonstrated that TB mortality was strikingly higher in the home-based compared to the facility-based DOT group under programmatic conditions. This is in contrast to three previous studies from Tanzania [9,20,21] which showed that mortality rates were lower or equal in the home-based compared to the facility-based DOT. A cluster randomized trial conducted in a rural district in Tanzania done in 2003, reported mortality to be lower in the home-based compared to facility-based DOT patient group [9]. However, the observed results may be due to the close supervision by community-based DOT observers and health care workers under trial conditions [9]. In 2009, shortly after the introduction of home-based DOT in Tanzania, an observational study conducted in several urban and rural settings reported a lower mortality in the PCT cohort (home-based DOT) compared to a historic cohort (facility-based DOT) [21]. As the authors argue, however, the difference could be explained by the more efficacious and shorter six-month regimen (rifampicin throughout regimen, a more potent anti-TB) during the PCT implementation compared to the eight months regimen in the historic cohort. Moreover, the availability of the treatment supporters in the PCT cohort was verified by the study team. Another observational study in the northern Tanzania reported no difference in TB mortality in home-based compared to facility-based DOT [20]. The higher mortality in facility-based DOT could be attributed to TB patients who were admitted at the national TB hospital, who may have been severely sick patients referred from other districts.

The higher mortality in the home-based DOT group could be explained by the fact that TB patients under home-based DOT had more frequently risk factors of death such as older age (>35 years), HIV infection and smear-negative TB. Similarly, a study from the Northern part of Tanzania showed that HIV-positive and smear-negative TB patients preferred home-based DOT [20]. In line with other studies from Tanzania [7,20], we found that the majority (more than 60%) of TB patients in Tanzania appear to prefer home-based compared to facility-based DOT. Home-based DOT could have been the likely choice because of convenience (e.g., older people who are less mobile or severely sick TB patients) and because patient related treatment costs are considerably lower [7,22]. Furthermore, adequate treatment supervision and adherence under home-based DOT may not have been guaranteed and implemented throughout the treatment period as stipulated in the guidelines [7,13,20], which could have caused the higher mortality in the home-based DOT group. The overall mortality during TB treatment in our study (7%) is comparable to previous studies in Tanzania (9%) [20], and also comparable to studies elsewhere in sub-Saharan Africa (5%) [23]. However, mortality was lower than in the neighboring country Malawi (22%) [24], and lower than the global average (16%) [1].

We found that patient characteristics such as older age, HIV infection, and smear-negative TB were risk factors for mortality. This is in line with previous reports from several countries in sub-Saharan Africa [25–29] and a systematic review [30]. Smear-negative TB is associated with a higher mortality possibly because smear-negative TB is more common in HIV-positive patients with severe immunosuppression [31]. Therefore, HIV-positive patients with severe immunosuppression may have developed the Immune Reconstitution Inflammatory Syndrome (IRIS), which is a consequence of the immune recovery after initiation of ART, and can result in death [32–35]. We further speculate that other life threatening opportunistic infections such as *Pneumocystis jirovecii* pneumonia (PCP) [36] may have contributed to the higher mortality in patients opting for home-based DOT. Furthermore, older age was also associated with higher mortality, consistent with several previous studies [24,27,28,37]. Older people often have additional comorbidities such as diabetes mellitus, hypertension and chronic obstructive pulmonary disease (COPD) which are independently associated with increased risk of mortality [30]. In addition, fatal hepatotoxicity due to anti-TB is more common among older patients [38]. Finally, we also found that TB retreatment was associated with increased mortality. Retreatment TB patients (treatment failures or treatment after loss to follow-up) may have undetected initial or acquired drug resistance while on TB treatment [36,39], which may contribute to a higher mortality [36]. Although the prevalence of multi-drug resistance is low in Tanzania (3.9% among retreatment category patients, 1.1% among new TB patients) [40], its contribution to the increased mortality cannot be ruled out.

Our study has several limitations. First, this was a retrospective analysis using routinely collected data. However, we analyzed a large dataset of TB patients notified to the National TB Programme over four years. Also the patients without the outcome of interest were equally distributed between home-based and facility-based DOT, and therefore we expect no bias of results. Therefore, these findings may reflect the “real life” situation under the programmatic conditions. Second, DOT preference by the patient is as recorded in the TB registers and we could not verify if patient remained with that choice throughout the TB treatment. However, the proportion of patients under home-based DOT in our study is very comparable to others previous studies done in Tanzania [7,13,20]; indicating that the data used in our study reflects the practice of DOT implementation in programmatic conditions. Third, additional factors such as socio-economic factors, causes of death, use of cotrimoxazole prophylaxis and anti-retroviral therapy (ART) and treatment supporter characteristics were not available from the routine dataset. However, we can reasonably assume that most of the TB patients included in our study were supported by their family members [13,21,22], and the use of CPT and ART initiation was given as per Tanzanian HIV and AIDS treatment guidelines [14]. Finally, we analyzed only data from an urban setting, and thus our findings may only be generalizable to other urban areas in sub-Saharan Africa.

In conclusion, our results demonstrated that patients opting for home-based DOT under programmatic conditions are more likely to have risk factors for mortality and have an increased mortality compared to facility-based DOT. Our findings suggest that there is a need for a risk assessment of patients at the time of TB diagnosis and a need for a careful monitoring of the implementation of DOT under programmatic conditions. The counseling and support of TB patients during TB treatment by trained health care workers may need to be improved, particularly in high-risk groups such as older people and those with comorbidities and co-infections.

Future research should focus on developing simple risk assessment tools based on evidence from prospective cohort studies [30,41] to identify patient and treatment supporter factors which may potentially influence mortality during TB treatment. In addition, operational research is needed to monitor the quality of treatment supervision and performance of DOT. Due to the limitations of current DOT strategies to assure proper drug intake [5,8], additional new innovative public health interventions such as medication monitors and mobile text message reminders [42] should also be considered in the transition phase to the “post-DOT” era [43]. Combined with shortened and simplified TB treatment regimens, this may further reduce mortality and improve clinical outcomes to ultimately meet the “End TB” WHO targets by 2035 [2].

## Supporting Information

S1 Table Baseline characteristics of included and excluded TB patients. (DOCX)

S2 Table Baseline characteristics of TB patients, stratified by HIV status. (DOCX)

S3 Table Baseline characteristics of patients who died and were alive after starting TB treatment, stratified by DOT category. (DOCX)
