# Supplementary material for: Home-Based and Facility-Based Directly Observed Therapy of Tuberculosis Treatment under Programmatic Conditions in Urban Tanzania
Source: PLoS One. 2016 Aug 11;11(8):e0161171. doi: 10.1371/journal.pone.0161171 (PMC4981322; doi:10.1371/journal.pone.0161171)
Supplement: S1 Table — (DOCX) [file pone.0161171.s001.docx]

**Home-based and facility-based Directly Observed Therapy of tuberculosis treatment under programmatic conditions in urban Tanzania**

**S1 Table**. **Patient characteristics of TB patients included and excluded in the study.**

| **Characteristics**  n (%) | **All**  **(n=4,866)**  **n (%)** | **Included**  **(n=4,472)**  **n (%)** | **Excluded (n=394)**  **n (%)** | **p-value** |
| --- | --- | --- | --- | --- |
| **Total, n (%)** | 4,866 (100) | 4,472 (91.9) | 394 (8.1) |  |
| **Sex** |  |  |  | 0.5 |
| Male | 2,960 (60.8) | 2,714 (60.7) | 246 (62.4) |  |
| Female | 1,906 (39.2) | 1,758 (39.3) | 148 (37.6) |  |
| **Age in years, median (IQR)** | 35 (27-44) | 35 (27-44) | 34 (28-44) | 0.9 |
| **Age groups, years** |  |  |  | 0.9 |
| 15-19 | 285 (5.9) | 267 (6.0) | 18 (4.6) |  |
| 20-24 | 546 (11.2) | 495 (11.1) | 51 (12.9) |  |
| 25-29 | 727 (14.9) | 670 (15.0) | 57 (14.5) |  |
| 30-34 | 825 (17.0) | 753 (16.8) | 72 (18.3) |  |
| 35-39 | 709 (14.6) | 652 (14.6) | 57 (14.5) |  |
| 40-44 | 573 (11.8) | 531 (11.9) | 42 (10.7) |  |
| 45-49 | 435 (8.9) | 400 (8.9) | 35 (8.9) |  |
| 50-54 | 283 (5.8) | 260 (5.8) | 23 (5.8) |  |
| ≥ 55 | 483 (9.9) | 444 (9.9) | 39 (9.9) |  |
| **HIV status** |  |  |  | 0.4 |
| Positive | 1,936 (39.8) | 1,786 (39.9) | 150 (38.1) |  |
| Negative | 2,597 (53.4) | 2,376 (53.1) | 221 (56.1) |  |
| Unknown | 333 (6.8) | 310 (6.9) | 23 (5.8) |  |
| **Site of disease** |  |  |  | 0.054 |
| PTB | 4,001 (82.2) | 3,663 (81.9) | 338 (85.8) |  |
| EPTB | 865 (17.8) | 809 (18.1) | 56 (14.2) |  |
| **Patient category** |  |  |  | 0.080 |
| New | 4,735 (97.3) | 4,357 (97.4) | 378 (95.9) |  |
| Retreatment | 131 (2.7) | 115 (2.6) | 16 (4.1) |  |
| **AFB smear results at diagnosis** |  |  |  | 0.052 |
| Smear-positive | 2,455 (50.5) | 2,235 (50.0) | 220 (55.8) |  |
| Smear-negative | 2,366 (48.2) | 2,197 (49.1) | 169 (42.9) |  |
| Unknown smear results | 45 (0.9) | 40 (0.9) | 5 (1.3) |  |

n (%), absolute number and column percentage; TB, Tuberculosis; PTB, Pulmonary Tuberculosis; EPTB, Extrapulmonary Tuberculosis; IQR,Inter Quartile Range.

We excluded 31 patients with unknown DOT preference, and 363 patients with the outcome “not evaluated” (“unknown” outcome or “transferred out”). Of the 31 patients with unknown DOT preference, only 2 patients died.
