# Supplementary material for: Home-Based and Facility-Based Directly Observed Therapy of Tuberculosis Treatment under Programmatic Conditions in Urban Tanzania
Source: PLoS One. 2016 Aug 11;11(8):e0161171. doi: 10.1371/journal.pone.0161171 (PMC4981322; doi:10.1371/journal.pone.0161171)
Supplement: S2 Table — (DOCX) [file pone.0161171.s002.docx]

**Home-based and facility-based Directly Observed Therapy of tuberculosis treatment under programmatic conditions in urban Tanzania**

**S2 Table**. **Patient characteristics of TB patients stratified by HIV status.**

| Characteristic | HIV-positive  (n=1,936) | HIV-negative  (n=2,597) | p-value | HIV status unknown  n =2597 | All |
| --- | --- | --- | --- | --- | --- |
| Sex |  |  | <0.001 |  |  |
| Male | 970 (50.1) | 1,791 (69.0) |  | 199 (59.8) | 2,960 (60.8) |
| Female | 966 (49.9) | 806 (31.0) |  | 134 (40.2) | 1,772 (39.1) |
| Age in years, median (IQR) | 38 (31-45) | 32 (25-42) | <0.001 |  | 35 (27-44) |
| Site of disease |  |  | 0.022 |  |  |
| PTB | 1,570 (81.1) | 2,174 (83.7) |  | 257 (77.2) | 4,001 (82.2) |
| EPTB | 366 (18.9) | 423 (16.3) |  | 76 (22.8) | 865 (17.8) |
| Category |  |  | 0.1 |  |  |
| New | 1,877 (97.0) | 2,536 (97.7) |  | 322 (96.7) | 4,735 (97.3) |
| Retreatment | 59 (3.0) | 61 (2.3) |  | 11 (3.3) | 131 (2.7) |
| TB treatment outcomes |  |  | <0.001 |  |  |
| Treatment success | 1549 (80.0) | 2198 (84.6) |  |  | 3747 (82.6) |
| Cured | 577 (29.8) | 1083 (41.7) |  | 87 (26.1) | 1,747 (35.0) |
| Treatment completed | 972 (50.2) | 1115 (42.9) |  | 196 (58.9) | 2,283 (46.9) |
| Died | 195 (10.1) | 133 (5.1) |  | 19 (5.7) | 347 (7.1) |
| Loss to follow-up | 43 (2.2) | 44 (1.7) |  | 11 (3.3) | 87 (1.9) |
| Treatment failed | 7 (0.4) | 15 (0.6) |  | 2 (0.6) | 24 (0.5) |
| Not evaluated | 142 (7.3) | 207 (8.0) |  | 18 (5.4) | 363 (7.5) |
| AFB smear results at TB diagnosis |  |  | <0.001 |  |  |
| Smear-positive | 839 (43.3) | 1,468 (56.5) |  | 148 (44.4) | 2,307 (50.9) |
| Smear-negative | 1,065 (55.0) | 1,119 (43.1) |  | 182 (54.7) | 2,184 (48.2) |
| Unknown smear results | 32 (1.7) | 10 (0.4) |  | 3 (0.9) | 42 (0.9) |
| DOT preference |  |  | 0.003 |  |  |
| Home-based | 1,485 (76.7) | 1,874 (72.2) |  | 234 (70.3) | 3,359 (74.1) |
| Facility-based | 442 (22.8) | 708 (27.3) |  | 92 (27.6) | 1,150 (25.4) |
| Unknown | 9 (0.5) | 15 (0.6) |  | 7 (2.1) | 24 (0.5) |

n (%), absolute number and column percentage; TB, Tuberculosis; PTB, Pulmonary Tuberculosis; EPTB, Extrapulmonary Tuberculosis; IQR, Inter Quartile Range; DOT, Directly Observed Treatment

HIV-positive and HIV-negative patients were compared using the Chi-square test for categorical variables and the nonparametric Wilcrox-ranksum test for continuous variables
