# Supplementary material for: Home-Based and Facility-Based Directly Observed Therapy of Tuberculosis Treatment under Programmatic Conditions in Urban Tanzania
Source: PLoS One. 2016 Aug 11;11(8):e0161171. doi: 10.1371/journal.pone.0161171 (PMC4981322; doi:10.1371/journal.pone.0161171)
Supplement: S3 Table — (DOCX) [file pone.0161171.s003.docx]

**Home-based and facility-based Directly Observed Therapy of tuberculosis treatment under programmatic conditions in urban Tanzania**

**S3 Table.** **Patient characteristics of TB patients who died and were alive during TB treatment, stratified by the preference of DOT.**

|  | All patients  (n=4,472) | | Home-based  (n=3,320) | | |  | Facility-based  (n=1,152) | | |
| --- | --- | --- | --- | --- | --- | --- | --- | --- | --- |
|  |  | | **Dead** | **Alive** | **p-value** |  | **Dead** | **Alive** | **p-value** |
| Sex |  | |  |  | 0.2 |  |  |  | 0.2 |
| Male | 2,714 (60.7) | | 160 (54.2) | 1,759 (58.1) |  |  | 39 (78.0) | 756 (68.6) |  |
| Female | 1,758 (39.3) | | 135 (45.8) | 1,266 (41.9) |  |  | 11 (22.0) | 346 (31.4) |  |
| Age in years, median (IQR) | 35 (27-44) | | 40 (32-49) | 35 (27-45) | <0.001 |  | 35 (26-42) | 34 (28-41) | 0.8 |
| Age groups in years | | |  |  | <0.001 |  |  |  | 0.09 |
| 15-19 | | 267 (6.0) | 13 (4.4) | 214 (7.1) |  |  | 2 (2.0) | 38 (3.4) |  |
| 20-24 | | 495 (11.1) | 15 (5.1) | 336 (11.1) |  |  | 3 (6.0) | 141 (12.8) |  |
| 25-29 | | 670 (15.0) | 19 (6.4) | 465 (15.4) |  |  | 12 (24.0) | 174 (15.8) |  |
| 30-34 | | 753 (16.8) | 52 (17.6) | 473 (15.6) |  |  | 6 (12.0) | 222 (20.1) |  |
| 35-39 | | 652 (14.6) | 44 (14.9) | 436 (14.4) |  |  | 12 (24.0) | 160 (14.5) |  |
| 40-44 | | 531 (11.9) | 43 (14.6) | 332 (11.0) |  |  | 6 (12.0) | 150 (13.6) |  |
| 45-49 | | 400 (8.9) | 40 (13.6) | 261 (8.6) |  |  | 1 (2.0) | 98 (8.9) |  |
| 50-54 | | 260 (5.8) | 28 (9.5) | 174 (5.8) |  |  | 5 (10.0) | 53 (4.8) |  |
| ≥ 55 | | 444 (9.9) | 41 (13.9) | 334 (11.0) |  |  | 3 (6.0) | 66 (6.0) |  |
| HIV status | |  |  |  | <0.001 |  |  |  | 0.2 |
| Positive | | 1,786 (39.9) | 175 (59.3) | 1,197 (39.6) |  |  | 19 (38.0) | 395 (35.8) |  |
| Negative | | 2,376 (53.1) | 108 (36.6) | 1,620 (53.6) |  |  | 24 (48.0) | 624 (56.6) |  |
| Unknown | | 310 (6.9) | 12 (4.1) | 208 (6.9) |  |  | 7 (14.0) | 83 (7.5) |  |
| Site of disease | |  |  |  | 0.007 |  |  |  | 0.6 |
| PTB | | 3,663 (81.9) | 218 (73.9) | 2,434 (80.5) |  |  | 45 (90.0) | 966 (87.7) |  |
| EPTB | | 809 (18.1) | 77 (26.1) | 591 (19.5) |  |  | 5 (10.0) | 136 (12.3) |  |
| Patient category | |  |  |  | <0.001 |  |  |  | 0.003 |
| New | | 4,357 (97.4) | 284 (96.3) | 3,004 (99.3) |  |  | 41 (82.0) | 1,028 (93.3) |  |
| Retreatment | | 115 (2.6) | 11 (3.7) | 21 (0.7) |  |  | 9 (18.0) | 74 (6.7) |  |
| AFB smear results at diagnosis | | |  |  | <0.001 |  |  |  | 0.6 |
| Smear-positive | | 2,235 (50.0) | 83 (28.1) | 1,479 (48.9) |  |  | 26 (52.0) | 647 (58.7) |  |
| Smear-negative | | 2,197 (49.1) | 205 (69.5) | 1,516 (50.1) |  |  | 24 (48.0) | 452 (41.0) |  |
| Unknown | | 7 (2.0) | 7 (2.4) | 30 (1.0) |  |  | 0 (0.0) | 3 (0.3) |  |

HIV-positive and HIV-negative were compared using pearson chi-square test for categorical variables and nonparametric Mann-Whitney test for continous variable

*n (%), absolute number and column percentage; TB, Tuberculosis; PTB, Pulmonary Tuberculosis; EPTB, Extrapulmonary Tuberculosis; IQR, Inter Quartile Range; DOT, Directly Observed Therapy
